# Harnessing Colon Chip Technology to Identify Commensal Bacteria That Promote Host Tolerance to Infection

**DOI:** 10.3389/fcimb.2021.638014

**Published:** 2021-03-12

**Authors:** Francesca S. Gazzaniga, Diogo M. Camacho, Meng Wu, Matheus F. Silva Palazzo, Alexandre L. M. Dinis, Francis N. Grafton, Mark J. Cartwright, Michael Super, Dennis L. Kasper, Donald E. Ingber

**Affiliations:** ^1^ Wyss Institute for Biologically Inspired Engineering, Harvard University, Boston, MA, United States; ^2^ Department of Immunology, Harvard Medical School, Boston, MA, United States; ^3^ Biophysics Institute Carlos Chagas Filho, Federal University of Rio de Janeiro, Rio de Janeiro, Brazil; ^4^ Vascular Biology Program and Department of Surgery, Boston Children’s Hospital and Harvard Medical School, Boston, MA, United States; ^5^ Harvard John A. Paulson School of Engineering and Applied Sciences, Harvard University, Cambridge, MA, United States

**Keywords:** microfluidic, microbiome, tolerance, gut, intestine, infection

## Abstract

Commensal bacteria within the gut microbiome contribute to development of host tolerance to infection, however, identifying specific microbes responsible for this response is difficult. Here we describe methods for developing microfluidic organ-on-a-chip models of small and large intestine lined with epithelial cells isolated from duodenal, jejunal, ileal, or colon organoids derived from wild type or transgenic mice. To focus on host-microbiome interactions, we carried out studies with the mouse Colon Chip and demonstrated that it can support co-culture with living gut microbiome and enable assessment of effects on epithelial adhesion, tight junctions, barrier function, mucus production, and cytokine release. Moreover, infection of the Colon Chips with the pathogenic bacterium, *Salmonella typhimurium*, resulted in epithelial detachment, decreased tight junction staining, and increased release of chemokines (CXCL1, CXCL2, and CCL20) that closely mimicked changes previously seen in mice. Symbiosis between microbiome bacteria and the intestinal epithelium was also recapitulated by populating Colon Chips with complex living mouse or human microbiome. By taking advantage of differences in the composition between complex microbiome samples cultured on each chip using 16s sequencing, we were able to identify *Enterococcus faecium* as a positive contributor to host tolerance, confirming past findings obtained in mouse experiments. Thus, mouse Intestine Chips may represent new experimental *in vitro* platforms for identifying particular bacterial strains that modulate host response to pathogens, as well as for investigating the cellular and molecular basis of host-microbe interactions.

## Introduction

The epithelium lining the intestine is an essential barrier that protects our bodies from the trillions of microbes within the microbiome that resides within its lumen. The intestinal epithelium regulates this critical barrier by integrating signals from commensal bacteria within the microbiome with cues from immune cells in the lamina propria, such that a state of host tolerance develops which enables host-microbiome symbiosis. Maintaining this state of tolerance in which microbes and the host co-exist without causing damage to each other is essential to protect against a wide variety of diseases. Great advances in our understanding of host-microbiome relations has been made through use of studies in mice where both the composition of the microbiota and host immune cell regulatory responses have been well characterized (Chung et al., 2012; Hooper et al., 2012; Geva-Zatorsky et al., 2017). However, it is extremely difficult to identify specific cellular and molecular contributors to host tolerance using *in vivo* experiments alone. Recently developed organ-on-a-chip (Organ Chip) microfluidic culture technology offers a way to recapitulate organ-level structures and functions *in vitro*. Thus, in the present study, we set out to explore if Organ Chip technology could be leveraged to develop an *in vitro* model of the mouse intestinal barrier in the colon that can be used to study host-microbiome interactions that contribute to tolerance development.

Studies in mice suggest that gut bacteria can protect from infection by several mechanisms. For example, differences in gut microbiota contribute to species-specific differences in resistance to *S. typhimurium* infection where the half maximal infectious dose (ID_50_) is more than three logs higher in mice than in humans, unless the mice are pretreated orally with streptomycin to alter the microbiome (Palmer and Slauch, 2017). Furthermore, when gnotobiotic mice that lack a microbiome are recolonized with healthy mouse microbiota, they become more resistant to *S. typhimurium* infection than when colonized with healthy human microbiota (Chung et al., 2012).

In addition to colonization resistance, the microbiome can also protect from infection by inducing host tolerance. *E. faecium*, a bacterium found in healthy human stool, promotes tolerance to *S. typhimurium* in mice as well as in *C. elegans* (Pedicord et al., 2016; Rangan et al., 2016). *S. typhimurium* colonizes the intestine of mice containing *E. faecium*, but disease severity is limited due to thickening of the mucus layer, which prevents this potential pathogen from accessing the gut epithelium. In other cases, gut bacteria prevent infection by impacting the host immune system. For instance, Type 3 Innate Lymphoid Cells (ILC3s) located in the intestinal lamina propria protect against infection by responding to microbial signals and releasing the cytokine interleukin-22 (IL22), which promotes production of antimicrobial peptides (Longman et al., 2014). With the rise of antibiotic resistance, understanding how microbes impact the gut epithelium to maintain a tolerance state is critical for developing new and more effective anti-infective therapeutics.

Microfluidic Organ Chips lined by living cells isolated from host tissues can be used to recapitulate organ level behaviors with high fidelity *in vitro*, and to interrogate cellular interactions with great precision in an organ-level context (Kim et al., 2012; Bhatia and Ingber, 2014; Benam et al., 2016; Kasendra et al., 2018). Human Intestine Chips have been created that are lined with primary epithelial cells isolated from patient-derived organoids (Kim et al., 2012; Kasendra et al., 2018; Sontheimer-Phelps et al., 2020), which provide multiple advantages over organoid culture alone. In contrast to organoids, Organ Chips enable culture of complex living microbiome in direct contact a differentiated intestinal epithelium and its overlying mucus layer while experiencing dynamic flow and peristalsis-like motions, in addition to providing direct access to the apical and basal compartments so that barrier, transport, and absorptive functions can be measured (Bhatia and Ingber, 2014; Kasendra et al., 2018; Tovaglieri et al., 2019). Individual cell types also can be added or removed in different combinations to Organ Chips to pinpoint the directionality of cell-cell and tissue-tissue interactions. For example, human Colon Chips were recently used to investigate how microbiota metabolites from mouse *versus* human feces impact susceptibility to enterohemorrhagic *E. coli* infection (Tovaglieri et al., 2019). This system replicated species-specific findings that mouse microbial metabolites provided better protection from infection than those from human microbiome, and led to the identification of human microbiome metabolites that increase susceptibility to infection (Tovaglieri et al., 2019).

While most Organ Chips have used human cells to mimic human physiology (Benam et al., 2016; Jalili-Firoozinezhad et al., 2019), chips created with mouse intestinal epithelial cells would provide the benefit of being able to directly compare chip responses to results of prior animal studies, while also providing the ability to isolate and add physiologically relevant cells that are often difficult to obtain from human biopsies. Here we describe methods for developing mouse Intestine Chips lined by epithelial cells isolated from duodenum, ileum, jejunum, or colon. We also show that the Colon Chip supports co-culture of complex gut microbiome with differentiated intestinal epithelium and show that this experimental model can be used to investigate specific host-microbe interactions under controlled conditions *in vitro*.

## Materials and Methods

### Isolation of Mouse Intestinal Crypts

C57/Bl6 WT or Kaede mice were sacrificed by CO_2_ asphyxiation followed by cervical dislocation and the large and small intestine was removed. The small intestine was separated into three segments: duodenum, jejunum, ileum, and colon. All intestinal crypts were isolated based on the Stem Cell Technologies protocol. Intestinal segments were flushed with cold PBS and cut lengthwise and then cut into 2 mm pieces and placed into a 50 ml conical tube with 10 ml cold PBS. Using a pre-wetted 10 ml pipette, intestine pieces were pipetted vigorously up and down three times. After intestinal pieces settled to the bottom of the tube, the supernatant was removed. This procedure was repeated until the supernatant was clear (10–20 times). Clear supernatant was removed and intestinal pieces were resuspended in 25 ml of Gentle Cell Dissociation Reagent (Stem Cell Technologies, 07174). Tubes were incubated at room temperature on a rocking platform for 20 min. Tubes were removed from the rocking platform and intestinal pieces were left to settle to the bottom of the tube. Once the pieces settled, the supernatant was removed. Intestinal pieces were vigorously pipetted up and down three times in 10 ml of cold PBS + 0.1% BSA and left to settle on the bottom of the tube. Supernatant was collected, passed through a 70 micron filter, and labeled as fraction 1. This step was repeated for a total of four fractions. Fractions were spun down at 290 g for 5 min at 4°C. Supernatant was removed and fractions were resuspended in 10 ml cold PBS + 0.1% BSA. Then 1 ml of each fraction was placed in a 24-well dish and the quality of the crypts were assessed by an inverted microscope. The fraction with the most crypts and fewest single cells was chosen. Crypts were counted and spun down 290 g for 5 min at 4°C. Crypts were resuspended in growth factor reduced Matrigel (Corning, 356231) at 30 crypts per microliter. Fifty microliters were plated onto 24-well non-tissue culture treated plates (Costar 3738). Plates were placed in 37°C incubator for 15 min to allow for Matrigel to solidify. Five hundred microliters of mouse organoid culture media (Advanced DMEM/F12 (12634028 Thermo Fisher Scientific), 50% L-WRN (Wnt3a, R-spondin, Noggin) conditioned media (Miyoshi and Stappenbeck, 2013), 2 mM glutamax (35050061 Thermo Fisher Scientific), 10 mM HEPES (15630080 Thermo Fisher Scientific), 50 ng/ml murine epidermal growth factor (Invitrogen PMG8043), N2 supplement (Invitrogen, 17502-048), B27 supplement (Invitrogen 17504-044), 1 mM N-Acetylcystine (Sigma Aldrich A9165-5G), 100 μg/ml Primocin (Invivogen ant-pm-1) plus 10 uM Y-27632 (Sigma Aldrich Y0503) was placed on top of the Matrigel domes. Media was changed three times a week and Y27632 was only added the day of splitting.

### Mouse Intestine Chip Cultures

The mouse colon chips use the same chip design, membrane activation, membrane coating, organoid harvesting, and seeding as previously described (Kasendra et al., 2018; Jalili-Firoozinezhad et al., 2019; Li et al., 2019; Sontheimer-Phelps et al., 2020). Briefly, microfluidic chips composed of PDMS and two parallel microchannels separated by a porous membrane were purchased from Emulate, Inc (CHIP-S1 Stretchable Chip, RE00001024 Basic Research Kit; Emulate, Inc). The inner surfaces of both channels were activated with 0.5 mg/ml sulfo-SANPAH solution (A35395, Thermo Fisher Scientific) as in (Li et al., 2019). The inner surfaces of both channels and membrane were coated with 200 ug/ml rat tail collagen type I (354236, Corning) and 4% Matrigel (Corning, 356231) as previously described (Kasendra et al., 2018). Mouse intestine organoids (duodenum, jejunum, ileum, colon) were maintained in 50 μl of Matrigel in a 24-well dish with 500 ul mouse organoid media and split at 1:4 ratio once per week. Two days before chip seeding, organoids were split as previously described (Sontheimer-Phelps et al., 2020). Organoids were isolated from Matrigel and fragmented as previously described (Sontheimer-Phelps et al., 2020). Organoids were resuspended in mouse organoid medium supplemented with 10 μM/L Y-27632 at 6 × 10^6^ cells/ml. The basal channel of extracellular matrix coated chips was filled with 50 μl of mouse organoid media supplemented with 10 μM/L Y-27632 and plugged with 200 ul filter tips at the outlet and inlet. Then 35 μl of disrupted organoids were seeded onto the top channel and the outlet and inlets were plugged with 200 ul filter tips. Chips were incubated overnight at 37C in 5% CO_2_ to promote cell adhesion. The following day, the apical channel was washed with 100 ul of mouse organoid media at a time until all unattached cells were removed. The chips were placed in the anaerobic farms and connected to peristaltic pumps as previously described (Jalili-Firoozinezhad et al., 2019). Duodenum, jejunum, ileum, or colon chips were perfused with mouse organoid media on the apical and basal channels at 1 μl/min. Media was added to reservoirs every other day. Mouse intestine chips formed confluent monolayers 1 week after culture. After monolayer formation, anaerobic gas (5% CO_2_ 95% N_2_) was perfused through the apical channel of the anaerobic farm as previously described (Jalili-Firoozinezhad et al., 2019).

### Immunofluorescent Microscopy

Both channels of mouse colon chips were washed with 100 μl of PBS and then fixed with 50 μl of 4% PFA for 15 min at room temperature. Chips were washed with 100 μl of PBS and blocked and permeabilized with 100 μl of 0.1% Triton X-100 and 5% BSA in PBS for 1 h at room temperature. Samples were stained overnight with the following primary antibodies diluted 1:100 in 5% BSA in PBS at 4C: anti-MUC2 (Santa Cruz Biotechnology sc-15334), anti-ZO-1 (Santa Cruz Biotechnology sc-33725), anti-ChrA (Santa Cruz Biotechnology sc-1488), anti-phalloidin 647 (Thermo-Fisher A22287). The following day, chips were washed with 100 ul PBS per channel and stained with the following secondary antibodies diluted 1:100 in 5% BSA in PBS for 2 h at room temperature: goat anti-rat Alexa Fluor 488 (Thermo Fisher Scientific A11006), donkey anti-rabbit Alexa Fluor 647 (Thermo Fisher Scientific A31573), donkey anti-goat Alexa Fluor 555 (Thermo Fisher Scientific A21432). Chips were washed with 100 ul of PBS three times. During the second wash, chips were incubated with 5 μg/ml DAPI (Thermo Fisher Scientific D1306) for 5 min at room temperature. Chips were imaged with a laser scanning confocal microscope (Leica SP5 X MP DMI-6000). Images were processed in FIJI2 and nuclei coverage and ZO-1 intensity was analyzed using FIJI2. For imaging Kaede chips, Kaede chips were photoconverted by shining a 405 nm laser for 30 s.

### Cytokines/Chemokines Analysis

Cytokine and chemokine levels were measured in the basal outflow of mouse intestine chips using a custom MSD U-plex Assay (Meso Scale Diagnostics) and the mouse CXCL1/KC DuoSet ELISA (R&D Systems DY453) according to the manufacturers’ instructions.

### Generation and Culture of *Salmonella typhimurium mCherry*

To generate *S. typhimurium-mCherry*, the plasmid pAW83-mCherry was isolated with a miniprep kit (Qiagen) and then transfected into electrocompetent *Salmonella enterica serovar typhimurium* strain SL1344 through the use of electroporation. Transfected cells were grown on selective LB agar with 100 μg/ml carbenicillin, and a single colony was selected to produce 15% glycerol stocks for −80°C storage as well as confirmation of mCherry expression.

To establish long-lived quantified stocks for infection, the *S. typhimurium-mCherry* was grown to exponential phase in a large volume of LB broth with 100 μg/ml carbenicillin. The exponential growth was then centrifuged, the pellet was washed once, and then reconstituted in ice cold sterile saline/dextrose (5% dextrose and 0.45% sodium chloride) with 15% glycerol to generate an optical density (OD) value equivalent to 1e9 CFU/ml. It was then split across several small aliquots stored at −80°C, and an aliquot was plated after freeze-thaw to confirm CFU accuracy.

### *E. faecium* Culture

*E. faecium* isolated from Hmb stock was grown overnight at 37°C in aerobic conditions in brain heart infusion media (B11059 BD Biosciences). *E. faecium* was pelleted at 5,000 g, washed once in DMEM, and resuspended in antibiotic free bacterial mouse organoid media (mouse organoid media + 1 mg/ml pectin, 1 mg/ml mucin, 5 μg/ml Hemin, 0.5 μg/ml Vitamin K1) at 1 × 10^6^ CFU/ml.

### Bacterial Colonization of Chips

Twenty-four hours before bacterial colonization, reservoirs were washed with PBS and antibiotic free mouse organoid media and antibiotic free bacterial mouse organoid media was added to the basal and apical channels, respectively. On the day of colonization, anaerobic farms were moved to an anaerobic chamber. Basal channels were plugged with 200 ul filter tips. Fifty μl of 1 × 10^6^ *S. typhimurium* or *E. faecium*, or 50 μl of 1:100 human microbiome (Hmb) or mouse microbiome (Mmb) stock [generated as in (Jalili-Firoozinezhad et al., 2019)] was seeded into the apical channel. Anaerobic farms were reconnected to pumps and left static for 30 min before media was perfused at 1 μl/min. In some experiments*, E. faecium*, Hmb, or Mmb, was perfused in colon chips for 16 h before infection with *S. typhimurium*. Chips were flushed at 50 μl/min for 2 min and outflow was plated in eight 10-fold serial dilutions onto Bile Esculin Agar and Xylose Lysine Deoxycholate plates for *E. faecium* and *S. typhimurium* samples or frozen at −80°C for 16S sequencing.

### 16S rRNA Profiling of the Microbiota

Frozen chip outflow was either sent to Diversigen for 16S sequencing (**Figure 3A** and **Figure S6**) and analysis (**Figure 3A**) or processed and analyzed in house using the same primers as Diversigen (**Figures 4A, B**). When processed in house, bacterial DNA was extracted using QIAamp Fast DNA Stool Mini Kit (Qiagen 51604). Purified DNA was quantified by Qubit dsDNA HS Assay (Invitrogen) and normalized. The V4 region of 16S rRNA gene was amplified with primers 515F and 806R under the previously described conditions (Caporaso et al., 2012), and ~390-bp amplicons were purified and quantified by Qubit dsDNA HS Assay and combined with equal mass to make a pooled library. The pooled library was then subjected to multiplex sequencing (Illumina MiSeq, 251 nt x 2 pair-end reads with 12 nt index reads). Raw sequencing data were processed with QIIME2 pipelines (Caporaso et al., 2012). In brief, raw sequencing data were imported to QIIME2 and demultiplexed, then DADA2 were used for sequence quality control and feature table construction. The feature table were further used for alpha and beta diversity analysis, as well as taxonomic analysis.

## Results

### Mouse Intestine Chips Can Be Generated From Wild Type or Transgenic Mice

**Figure 1:**
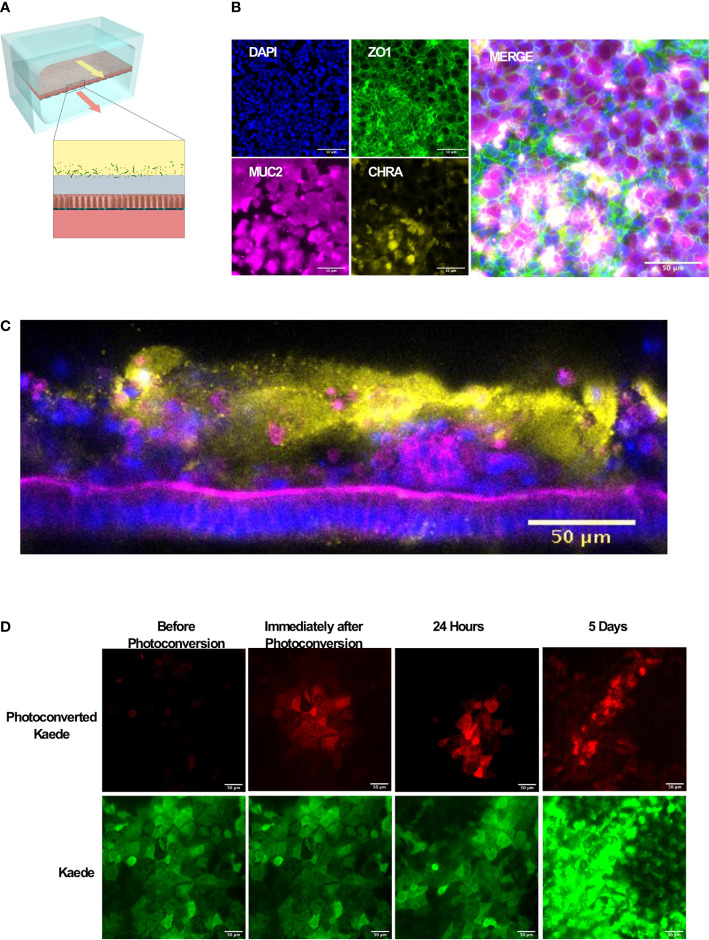
Microfluidic mouse Colon Chips form tight junctions, multiple cell types, and a mucus layer. **(A)** Diagram of a two-channel microfluidic Organ Chip with epithelial cells on top of a porous membrane separating the channels, and bacteria are cultured above the epithelium under a hypoxia gradient that is created by flowing oxygenated medium through the basal channel while maintaining the entire chip in an anaerobic chamber filled with carbon dioxide and nitrogen gas. **(B)** Immunofluorescence microscopic images showing mouse Colon Chip lined by an intestinal epithelium containing tight junctions (ZO-1, green) and goblet cells (MUC2, magenta), and enteroendocrine cells (CHR A, yellow); nuclei are stained with DAPI (blue). Images taken with 25× objective. **(C)** Cross section of mouse Colon Chip showing the polarized epithelium stained for F-actin (magenta) that concentrates at the apical brush border and for MUC2 (yellow), which appears in the overlying mucus layer; nuclei are stained with DAPI (blue). **(D)** Immunofluorescence top-down view showing that mouse Colon Chips form monolayers with cells isolated from organoids derived from Kaede mouse (green), which can be used to track cell divisions. A section of the chip was photoconverted with 405 nm light (red) and imaged daily. Dilution of red signal to green indicates cell turnover over time. Images taken with 25× objective.

To develop mouse Intestine Chips, we generated organoids from the colon of wild type (WT) C57/Bl6 mice or transgenic Kaede mice that express a photoconvertible GFP driven by the actin promoter that can be used to track cell divisions and migration when activated with 405 nm light (Morton et al., 2014). Organoids that were prepared as previously described (Sato et al., 2009; Sato et al., 2011; Miyoshi and Stappenbeck, 2013) were enzymatically disrupted and physically dissociated before being seeded on the upper surface of the extracellular matrix (ECM)-coated porous membrane that separates the two parallel microfluidic channels of the Organ Chip (**Figure 1A**). Organoid culture medium was perfused through both channels and a confluent monolayer was observed covering the porous membrane by 1 week of culture in both WT and Kaede chips, and similar results were obtained when we used intestinal epithelial cells isolated from organoids derived from mouse duodenum, jejunum, and ileum (**Supplementary Figures S1A–D**).

We focused here on Colon Chips to study host-microbiome interactions because this region of the intestine harbors the most complex microbiome with the highest total bacterial load (Mowat and Agace, 2014). Immunofluorescence microscopic analysis confirmed that the epithelial monolayer forms a continuous network of ZO-1-containing tight junctions and contains MUC2-positive goblet cells as well as enteroendocrine cells that stained positively for chromogranin A staining when viewed from above (**Figure 1B**). Measurements of intestinal barrier function over days 7–9 by quantifying the apparent permeability (P_app_) of Cascade Blue (550 Da) confirmed that barrier integrity is maintained for at least 9 days (**Supplementary Figure S2**), as previously demonstrated in human Colon Chips (Sontheimer-Phelps et al., 2020). Z stack visualization (**Supplementary Movie S1**) and immunostaining of vertical cross sections through the chips (**Figure 1C**) revealed a polarized epithelium with basal nuclei, apical junctions enriched for F-actin, and MUC2-staining droplets in the apical regions of the cells as well as a MUC2 rich mucus layer overlying the apical surfaces of the cells. Although all unattached cells were flushed away before flow was initiated, unattached cells could be detected in the apical mucus layer 1 week after starting flow suggesting that mouse Colon Chips shed epithelial cells while maintaining the epithelium, as observed in mouse colon *in vivo*.

To investigate if the intestinal epithelial cells are undergoing active turnover on-chip, sections of chips lined with the photosensitive GFP-expressing Kaede mouse cells were photoconverted and imaged daily. The dilution of the size of red area over time (**Figure 1D**, **Supplementary Figure S3**) coincided with an increase in number of red spots as the cells divided, breaking up the red area with new green cells (**Supplementary Figure S3**) within an otherwise continuous epithelium indicating the presence of constant epithelial turnover. Additionally, qPCR of Colon Chips and organoids after 8 days of culture in the same medium confirmed the expression of genes encoding the stem cell marker *Lgr5*, and the differentiation markers, *Muc2* and *ChrA* in all tissues (**Supplementary Figure S4**); however, there was significantly higher levels of *Muc2* and *ChrA* expression in the Colon Chips compared to the organoids. Although *Lgr5* was detectable in both chips and organoids, it was higher in organoids confirming previous reports that the microfluidic chips that experience fluid flow more efficiently promote intestinal differentiation (Kasendra et al., 2018; Sontheimer-Phelps et al., 2020).

### *S. typhimurium* Induces Epithelial Injury in Mouse Colon Chips

**Figure 2:**
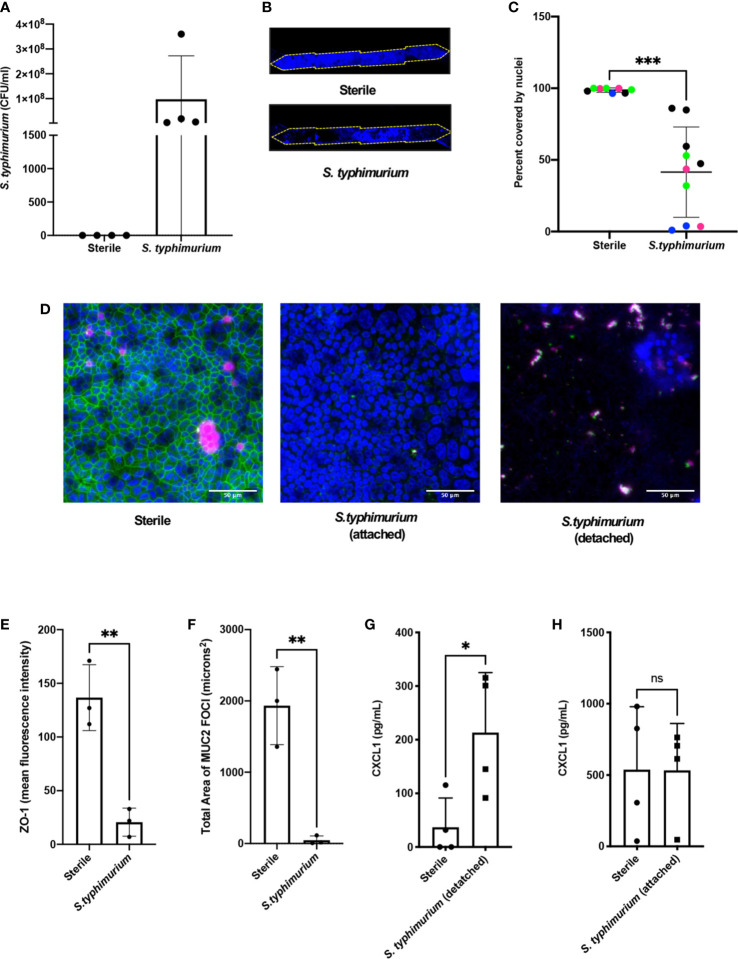
*S. typhimurium* induces epithelial damage in mouse Colon Chips. Chips were inoculated with 5 × 10^5^ CFU/ml and perfused for 24 h. **(A)** *S. typhimurium* CFU/ml was quantified by plating a fast flush (60 microliters/min) from chips 24 h after infection onto XLD plates and only detected in chips that were inoculated with *S. typhimurium.* Each dot represents a different chip; similar results were obtained in four different experiments. **(B)** Epithelial lesions induced by *S. typhimurium* infection were visualized by imaging chips in the DAPI channel at 5×, representative examples for Sterile and *S. typhimurium* chips are shown. Yellow dotted line outlines channel of chip. **(C)** Cell coverage was quantified by processing images using FIJI2 and calculating the area of the membrane surface covered by cells (shown as percent covered by nuclei). *S. typhimurium* induced significant epithelial cell detachment (Unpaired t test P= 0.0001); data shown are from four representative experiments (indicated by different colored dots). **(D)** Sterile and *S. typhimurium*-infected chips stained for DAPI (blue), ZO-1 (green), and MUC2 (magenta). Merged images indicate that *S. typhimurium* disrupts ZO-1 tight junction and reduces MUC2 expression before cell detachment occurs. Sterile and S. t. attached taken at 15 h. S.t. detached taken at 24 h. Images from sterile and S.t. chips 15 h after infection were analyzed in FIJI2 and differences in **(E)** ZO-1 intensity (mean fluorescence intensity) (Unpaired t-test P= 0.0038) **(F)** Area of MUC2 foci (microns^2^) (Unpaired t-test P = 0.004) were measured. CXCL1 levels in outflows of sterile chips *versus S. typhimurium* chips at **(G)** 24 h (Unpaired T-test P= 0.0297) or when chips were harvested at **(H)** 15 h after infection. *P < 0.05, **P < 0.01, ***P < 0.001. ns, not significant.

We then assessed if the mouse Colon Chips can be used to interrogate response to pathogens by comparing the responses of the epithelium to co-culture with a known mouse intestinal pathogen, *S. typhimurium* (Miyoshi and Stappenbeck, 2013; Jalili-Firoozinezhad et al., 2019; Tovaglieri et al., 2019), *versus* commensal bacteria from healthy mice. After being seeded in the apical channel of Colon Chips lined by confluent epithelium, mCherry-labeled *S. typhimurium* bacteria grew rapidly as visualized in fixed chips and by live cell imaging (**Supplementary Figures S5A, B** **)**. Quantification of the bacteria revealed that they increased in number by three logs within 24 h on-chip (**Figure 2A**), which is the same rate at which these bacteria grown when administered to germ free mice (Chung et al., 2012). Within 24 h of *S. typhimurium* infection, epithelial cell detachment could be detected by a decrease in the chip membrane surface area covered by nucleated cells (**Figures 2B, C** **)**. When chips were harvested prior to epithelial detachment at 15 h after infection instead of 24 h, ZO-1 intensity (**Figures 2D, E** **)** and the area covered by MUC2 foci were significantly reduced (**Figure 2F**) indicating that disruption of tight junctions and decreased mucus accumulation precede epithelial lesion formation.

To determine how cytokine and chemokine production in colon chips is impacted by *S. typhimurium* infection, the effluent of the basal channel of the chip was collected at 1.5, 3, 6, and 24 h after infection and assayed with a multiplex ELISA. We found that CXCL1 and CXCL2 (mouse homologs to IL8) increased at 24 h following *S. typhimurium* infection, whereas other cytokines were detected at much lower levels (**Supplementary Figures S6A–C**). Because intestinal epithelial cells secrete CXCL1 upon pathogen infection (Kagnoff, 2014), we measured CXCL1 as a metric for assessing bacterial pathogenicity of bacteria within the lumen of the engineered intestinal epithelium. These studies confirmed that *S. typhimurium* infection induced more than a 5-fold increase in CXCL1 production in the Colon Chip 24 h after infection (**Figure 2G**), but not 15 h after infection, when the monolayer is still intact (**Figure 2H**). RNA Seq analysis also confirmed that 24 genes were differentially expressed within 6 h after infection of the Colon Chips with *S. typhimurium*, with almost all (Jackson et al., 2018) genes showing an increase in expression (**Supplementary Figure S7**). Importantly, all of the genes that were over expressed by the intestinal epithelium in response to *S. typhimurium* infection on-chip (CXCL1, CXCL2, CCL20, and AW112010) behave similarly when mice are exposed to *S. typhimurium in vivo* (Sierro et al., 2001; Perkins et al., 2015; Jackson et al., 2018). These results indicate that mouse Colon Chips provide a physiologically relevant system to investigate *S. typhimurium* infection *in vitro*, and that epithelial detachment, decreased tight junction staining, and increased CXCL1 release may be used as metrics to assess bacterial pathogenicity on-chip.

### Co-Culture of Complex Human and Mouse Microbiota On-Chip

**Figure 3:**
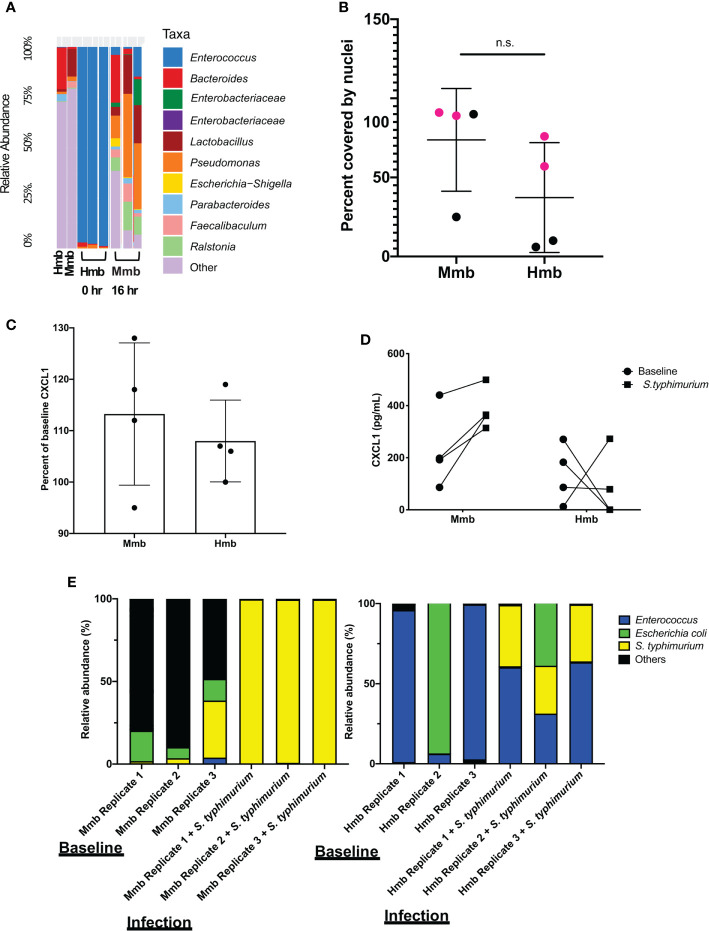
Colon Chips with microbiota can be used to select strains that protect against pathogen. **(A)** Microbiota derived from Hmb or Mmb mice was seeded onto mouse Colon Chips and 16S analysis was performed on seeding stocks and flushes after 16 h on chip. Relative abundance of the indicated genera reveals *Enterococcus* dominated the Hmb chips. **(B)** Culture surface areas covered by adherent nucleated cells varied between experiments and were not significantly different between Hmb or Mmb colonized chips. Data shown are from two different experiments (magenta *versus* black). **(C)** CXCL1 detected in basal outflows immediately before (baseline) or 16 h after colonization with Mmb or Hmb. Percent of baseline CXCL1 release shows no significant difference between colonization with Mmb *versus* Hmb; similar results were observed in four different experiments. **(D)** CXCL1 detected in basal outflows 16 h after colonization with Hmb or Mmb (baseline; solid circle), and 24 h after infection with *S. typhimurium* (*S. typhimurium;* solid square). Hmb colonization, but not Mmb colonization inhibited *S. typhimurium*-induced CXCL1 release. **(E)** 16S sequencing of bacteria in apical outflows collected 16 h after colonization with Mmb or Hmb (baseline) and 24 h after infection with *S. typhimurium* (infection) from three replicate chips. Analysis of the relative abundance of genera on chips infected with *S. typhimurium* for 24 h revealed *Enterococcus* from Hmb correlates with less *S. typhimurium* overgrowth. ns, not significant.

To determine whether the mouse Colon Chip can model symbiosis between microbiome bacteria and the intestinal epithelium, we seeded chips with healthy human microbiome (Hmb) or mouse microbiome (Mmb) that were previously shown to differ in their ability to promote intestinal barrier function both in gnotobiotic mice where these microbiota are maintained and passaged (2), and when cultured in human Colon Chips (Jalili-Firoozinezhad et al., 2019). When we compared the diversity between seeding stocks and the microbiome cultured on-chip for 40 h, we found that on chip both microbiota exhibited similar alpha diversity (a metric of within sample taxonomic unit diversity) (**Supplementary Figure S8**). However, the *Enterococcus* genus dominated the Hmb chips within 16 h of colonization whereas Mmb chips contained multiple genera with variation in abundances between chips (**Figure 3A**), which is likely due to the particulate nature of the dense microbiome sample when making dilutions and carrying out chip seeding. Compared to sterile chips without bacteria that were lined with nucleated epithelial cells covering from 97 to 100% of the surface area of the porous membrane, the presence of Hmb or Mmb induced variable levels of epithelial damage, with lesion areas covering from 9 to 94% of the membrane surface (**Figure 3B**). Intestinal epithelial cell secretion of CXCL1 upon microbiome colonization also varied between chips, but CXCL1 secretion was not significantly different after 16 h of colonization (**Figure 3C**). These results indicate that the mouse Colon Chips can model a symbiotic relationship between healthy microbes and the colon epithelium; however, seeding a complex population of microbes, as is present within the Hmb and Mmb samples, results in variation in both microbiome composition and epithelial damage between chips.

### Colon Chips With Microbiota Can Be Used to Select Strains That Protect Against Pathogen

We next asked whether the Colon Chips colonized with different microbiota can be used to identify particular bacterial strains that modulate host response to pathogens. Because the mouse microbiome protects against *S. typhimurium* infection in mice (Chung et al., 2012), we leveraged the variations in microbiome composition we observed between chips colonized with Hmb and Mmb to identify microbes that protect against *S. typhimurium* infection. When Colon Chips were seeded with Hmb or Mmb and infected with *S. typhimurium* 16 h later, Hmb colonization inhibited *S. typhimurium-*induced CXCL1 release **(** **Figure 3D** **)**. 16S sequencing revealed that *S. typhimurium* colonized both Hmb and Mmb chips (**Figure 3E**). However, while *S. typhimurium* outgrew all of the other microbiome bacteria in Mmb chips, this pathogen did not overwhelm chips colonized by Hmb microbiome (**Figure 3E**), which is dominated by *Enterococci* (**Figures 3A, E** **)**.

**Figure 4:**
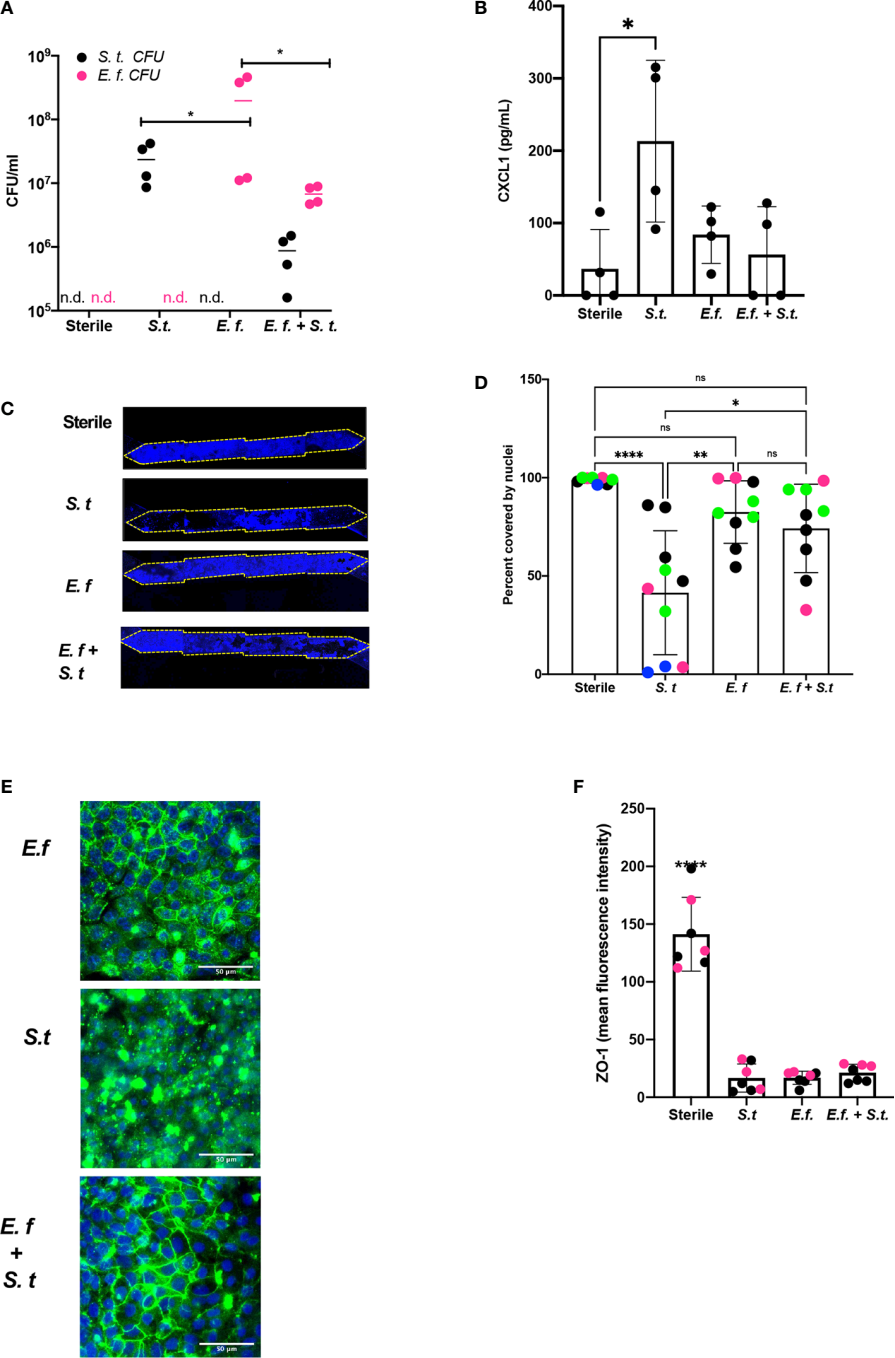
*Enterococcus faecium* protects colon epithelium from *S. typhimurium*-induced damage. Mouse Colon Chips were colonized with *E. faecium* for 16 h and infected with *S. typhimurium* 24 h. **(A)**. Chips were flushed for 2 min and flush was plated in serial dilutions on bile esculin agar (BEA) plates and xylose lysine deoxycholate (XLD) plates to selectively measure *E*. *faecium* (BEA, magenta) and *S. typhimurium* (XLD, black) growth. Both *E*. *faecium* and *S. typhimurium* grew when cultured alone in the Colon Chips, but their numbers were reduced by 1 log when both bacteria were cultured simultaneously (similar results were obtained in four different experiments). **(B)** Basal chip outflow was collected 24 h after infection with *S. typhimurium* and CXCL1 measured by ELISA. Representative example from four different experiments. (Significance measured by one way ANOVA and Bonferroni’s multiple comparisons test adjusted P = 0.031) **(C)** Epithelial layer integrity was visualized by imaging DAPI-stained chips at low magnification (5×); representative examples for Sterile, *S. typhimurium*, *E. faecium*, and *E. faecium + S. typhimurium* chips are shown. **(D)** Cell coverage of the culture surface was quantified by processing images using FIJI2 and calculated as percent surface area covered by nuclei. *S. typhimurium*, but not *E*. *faecium* induced significant epithelial cell detachment (one way ANOVA and Bonferroni’s multiple comparisons test. Adjusted P values **** < 0.0001, ** = 0.0015, * = 0.0148). Different colored dots indicate chips from four different experiments. **(E)** Representative immunofluorescent images of *E*. *faecium* monocolonized, *S. typhimurium* monocolonized, and *E. faecium + S. typhimurium* chips show *E*. *faecium* colonized chips maintain ZO-1 tight junction outlines and protects from *S. typhimurium*-induced disruption of ZO-1 tight junction outlines ZO-1 (green), DAPI (blue). **(F)** Quantification of ZO-1 mean fluorescence intensity in immunofluorescent images from sterile, *S. typhimurium*, *E*. *faecium*, and *S. typhimurium* + *E*. *faecium* colonized chips. Mean ZO-1 intensity was measured using FIJI2. Combination of two experiments (magenta *versus* black). Significance determined one way ANOVA and Bonferroni’s multiple comparisons test. Adjusted P value P < 0.0001. ns, not significant.

To assess whether a particular species of *Enterococcus* was responsible for protection against *S. typhimurium* overgrowth, we isolated *Enterococci* from our Hmb stock and identified a single *Enterococcus* species, *E. faecium* (**Supplementary Figure S9**), that exhibited this protective phenotype. When we colonized mouse Colon Chips with either *E. faecium* alone, *S. typhimurium* alone, or initially seeded with *E. faecium* and then infected with *S. typhimurium* 16 h later, both bacterial species grew in the chips, but when combined, neither species grew to the same level as monocolonized chips (**Figure 4A**). The presence of *E. faecium* alone did not induce CXCL1 release and, in fact, it prevented CXCL1 release induced by *S. typhimurium* (**Figure 4B**). When analyzed after 40 h of colonization with *E. faecium*, there was a mild, but not statistically significant, increase in lesion formation (0–37 *vs.* 0–2% of epithelial surface area) when compared to sterile chips (**Figures 4C, D** **)**. In contrast, *S. typhimurium* infection caused detachment of large regions of the epithelium and induced a significant increase in epithelial lesion formation (14–99% lesion area) by 24 h of infection (**Figures 4C, D** **)**. In chips colonized with *E. faecium*, tight junction outlines could be visualized, unlike in *S. typhimurium* infected chips without *E. faecium* **(** **Figure 4E** **)** suggesting that *E. faecium* colonization protects from tight junction disruption. However tight junction intensity in sterile chips was significantly brighter than in chips colonized with *E. faecium*, *S. typhimurium*, or *E. faecium + S. typhimurium* (**Figure 4F**) suggesting that although *E. faecium* does not induce significant epithelial lesions or ZO-1 distribution, it may impact its level of expression. These findings are consistent with past studies that showed *E. faecium* promotes tolerance to *S. typhimurium* infection in mice (Pedicord et al., 2016; Rangan et al., 2016), but that *E. faecium* overgrowth can also have harmful effects in hospitalized patients (Ubeda et al., 2010; Caballero et al., 2017). Our results further suggest that *E. faecium* may prevent *S. typhimurium* overgrowth as well. These findings are consistent with past studies that showed *E. faecium* promotes tolerance to *S. typhimurium* infection in mice (Pedicord et al., 2016; Rangan et al., 2016), and our results further suggest that *E. faecium* may prevent *S. typhimurium* overgrowth as well. These results show that mouse Colon Chips can be used to identify bacteria strains within complex microbiome that protect intestinal epithelium from pathogen overgrowth and that mouse colon chips are able to model the nuances of commensal pathobionts that have both protective and harmful effects.

## Discussion

Our results demonstrate that mouse Intestine Chips can be created with cells derived from organoids isolated from duodenum, jejunum, ileum, or colons from wild type mice or transgenic Kaede mice that express GFP in all of their cells (Morton et al., 2014). We also showed that both complex species-specific microbiota or individual types of bacteria can be cultured in the Colon Chips and that bacterial-specific effects on epithelial cell adhesion, tight junctions, barrier function, mucus production, and cytokine release can be quantified directly. When challenged with pathogenic *S. typhimurium*, the mouse Colon Chips responded differently depending on whether the lumen was also colonized with normal human or mouse complex microbiome. In the course of these studies analyzing individual differences in the microbiome composition of chips, we identified *E. faecium* isolated from human microbiome as a bacterium that promotes host tolerance to infection as measured by prevention of epithelial cell detachment; however, the presence of this bacterium also induced small regions of epithelial detachment highlighting the potential of a single type commensal bacterium to produce both beneficial and injurious properties. Mouse Intestine Chips may therefore be useful for future mechanistic studies designed to pinpoint interactions between specific microbes and the intestinal epithelium.

The mouse Intestine Chips we described provide multiple novel experimental approaches that could be useful for future studies on host-microbiome interactions. The generation of Intestine Chips using organoids from Kaede mice opens up the possibility top track cell movement and divisions, in addition to enabling Intestine Chips to be created from mice with a variety of genetic backgrounds. This ability to generate Organ Chips with different genetic backgrounds can be especially helpful when comparing sterile chips to chips colonized with microbiome, as re-deriving germ-free mice from different genetic backgrounds is difficult and expensive. Although we focused on colon chips in this study, chips generated from different parts of the intestine could be used in future studies to compare site-specific host responses to commensal and pathogenic bacteria. While this study focused on interactions the pathogenicity of gut bacteria in the context of living mouse intestinal epithelium, the endothelium and immune system also play critical roles in other host responses, including inflammation and tolerance to infection (Ayres and Schneider, 2012; Jamieson et al., 2013). Endothelium and circulating immune cells have been integrated into human Intestine Chips in the past and so this should be doable (Kasendra et al., 2018; Jalili-Firoozinezhad et al., 2019); however, this will require additional studies to optimize medium conditions, seeding densities, growth dynamics, and other factors that are necessary to co-culture multiple mouse cell types on-chip.

We also showed that fluorescent bacteria can be imaged in live and fixed chips enabling future visualization and mechanistic studies focused on interactions between bacteria and host epithelium as well as among different bacteria cell populations. In addition, we were able to use the Colon Chips to recapitulate mouse intestinal injury and cytokine responses to infection with pathogenic *S. typhimurium* bacteria *in vitro*, and to reconstitute host-microbiome symbiosis by populating Colon Chips with complex living mouse and human microbiome. Finally, we showed that differences in the composition of complex microbiome cultured on-chip can be leveraged to identify specific bacterial strains, such as *E. faecium*, which modulate host tolerance to infection by *S. tymphimurium*. Taken together, these findings suggest that the mouse Colon Chip provides a modular system in which specific bacteria and epithelial cells from different regions of the intestine can be co-cultured to interrogate a wide variety of host-microbe interactions.

In our studies focused on identification of bacteria that promote tolerance to infection, mouse Colon Chips were infected with the intestinal pathogen, *S. typhimurium*, as a positive control. In mice, *S. typhimurium* infection causes bloody diarrhea, weight loss, inflammation in the small intestine, cecum, and colon, extrusion of epithelial cells, sepsis, and death (Que and Hentges, 1985; Jepson et al., 1995; Knodler et al., 2010). *S. typhimurium* grew well in the Colon Chips and induced similar epithelial damage (e.g., tight junction disruption, cell detachment) as well as increases in cytokines that recruit immune cells such as CXCL1, CXCL2, CCL20, and a non-coding RNA, AW112010, all of which closely mimic those previously observed in infected mice *in vivo* (19–21). These results indicate that the mouse Colon Chip can be used to investigate epithelium-pathogen interactions and that *S. typhimurium* can be used as a positive control for epithelial damage in these Organ Chip models.

To determine whether the mouse Colon Chip can model a symbiotic relationship between bacteria and the epithelium, we colonized mouse chips with healthy human microbiome (Hmb) or healthy mouse microbiome (Mmb) isolated from Hmb or Mmb mice (Chung et al., 2012). While we have previously shown that Hmb promotes barrier function in human chips (Jalili-Firoozinezhad et al., 2019), we found here that the mouse Colon Chips exhibited more variable responses to colonization with complex microbiome. Given the variation in the microbiome composition we observed between chips, these differences might account for the different phenotypic responses observed in this study. Future studies exploring different microbiome compositions, in terms of bacterial strains or population diversity at the genus level, could be help to identify bacteria that promote epithelial integrity, which could have many applications in clinical settings and microbiome-based therapies. In addition to microbiome composition, the differences observed between different Hmb or Mmb chips also could be due to differences in bacterial load. Therefore, future studies should optimize seeding densities, flow rate, and colonization dynamics of specific microbial strains when investigating species-specific effects on the gut epithelium. Interestingly, even though Hmb and Mmb stock solutions contained similar levels of alpha diversity and the Hmb stock was the same as we previously reported (Jalili-Firoozinezhad et al., 2019), *Enterococcus* came to dominate the Hmb mouse gut chips whereas Mmb chips contained a more diverse set of species. Taken together, these results indicate that mouse Intestine Chips could be used in the future to investigate colonization dynamics between certain microbes or microbial consortia and host species.

We decided to use the variation between chips to our advantage to investigate how the microbiome affects the host response *S. typhimurium* infection. Regardless of the starting microbiome composition, *S. typhimurium* outcompeted the species in Mmb chips. *S. typhimurium* also grew in Hmb chips, but *Enterococcus* prevented *S. typhimurium* from dominating the microbiome. These results appear to conflict with a previous finding that Mmb protects from *S. typhimurium* better than Hmb (Chung et al., 2012). However, that study used Swiss Webster (SW) mice while our Hmb, and Mmb samples were isolated from stool obtained from C57/BL6 mice because this is the strain we isolated our organoids from as well. Furthermore, SW Hmb mice had a dampened immune response compared to Mmb mice suggesting that the Hmb caused more severe *S. typhimurium* infection *via* its effect on the immune system, as opposed to Hmb bacteria affecting *S. typhimurium* pathogenicity without involving immune cells. In support of our findings, several studies have shown that *E. faecium* promotes tolerance to *S. typhimurium* infection in mice and *C. elegans* by an immune independent mechanism (Pedicord et al., 2016; Rangan et al., 2016).

Because *Enterococcus* species such as *E. faecium* and *E. faecalis* can easily become resistant to vancomycin and cause sepsis in hospitalized patients, referred to as Vancomycin Resistant Enterococcus (VRE) disease, we sought to use the Colon Chips to: 1) determine if our *E. faecium* alone causes damage and 2) determine if our *E. faecium* can protect the epithelium from *S. typhimurium*-induced damage. Interestingly, we found that *E. faecium* protects from significant *S. typhimurium*-induced epithelial detachment, tight junction disruption, and CXCL1 induction, supporting findings in mice that *E. faecium* promotes a tolerance response to *S. typhimurium (* Rangan et al., 2016; Pedicord et al., 2016). However, colonization with *E. faecium* alone decreased tight junction intensity and appeared to produce some epithelial damage when analyzed across multiple chips, with lesions being observed in 0–37% of the epithelial surface in chips cultured with *E. faecium* alone vs. 0–2% in sterile chips and 14 to 96% in chips infected with *S. typhimurium*. Based on this observation, we suggest that *E. faecium* should not be used as a probiotic to prevent bacterial infections, and instead, understanding the bacterial molecules from *E. faecium* that protect against disease (Pedicord et al., 2016; Rangan et al., 2016) and the mechanisms of action, might lead to more useful therapies for patients.

## Supplemental Methods

### Permeability

To assess barrier permeability, Cascade Blue hydrazide Trilithium Salt (550 Daltons) (Invitrogen C3239) was resuspended in H_2_O at 10 mg/ml added to the apical channel at a final concentration of 50 μg/ml. Fluorescence intensity (380 nm/420 nm) from apical and basal channel effluents was measured using a multi-mode plate reader (BioTek NEO). Apparent permeability (Papp) was calculated using the following equation: Papp = (V_r_ * C_r_)/A * t * (C_d-out_ * V_d_ + C_r_ *V_r_)/(V_d_ + V_r_)). V_r_ is volume (ml) of receiving channel. V_d_ is volume (ml) of dosing channel. A is area of membrane separating channels (cm^2^). t is time of effluent collection (seconds). C_r_ is measured concentration (μg/ml) of Cascade Blue in receiving channel effluent. And C_d-out_ is measured concentration (μg/ml) of Cascade Blue in dosing channel effluent.

### RNA Isolation, Quantitative Reverse Transcription Polymerase Chain Reaction, and Microarray Analysis

For RNA isolation, each channel of chips or organoids in Matrigel in a 24-well dish were washed with PBS. Cells were harvested using RLT buffer + BME and RNA was extracted using the Qiagen RNAeasy mini kit (74106, Qiagen). cDNA was synthesized using 0.5 ug RNA, 500 ng of random primers (Invitrogen 48190-011), 0.5 mM dNTPs, 1X First-Strand Buffer, 5 mM DTT, and 100 U of Superscript III (Invitrogen 18080-044) according to the manufacturer’s instructions. cDNA was diluted 1:4 with water and amplified using LightCycler 480 DNA SYBR Green I Master (Roche Applied Science 04887352001) and run on the LightCycler 96 (Roche 05815916001) using the preset Sybr Amp Melt Curve. The second derivative max was used to identify transcript copy number and normalized to the housekeeping gene, β-actin. The following primers were used: Muc2 F ctgaccaagagcgaacac, Muc2 R catgactggaagcaactgga TGCTGGGGTTTTTGTGAATCTC, ChrA F ATCCTCTCTATCCTGCGACAC, ChrA R GGGCTCTGGTTCTCAAACACT, Lgr5 F CCTACTCGAAGACTTACCCAGT, Lgr5 R GCATTGGGGTGAATGATAGCA, β-actin F GGCTGTATTCCCCTCCATCG, β-actin R CCAGTTGGTAACAATGCCATGT. For microarray RNA was extracted as described above and sent to Advanced Biomedical Laboratories for Microarray sequencing using the mouse Clariom D array. All analyses were run in R with custom scripts. Differentially expressed genes with a false discovery rate of q < 0.05 were plotted.

### Live Imaging of Mouse Colon Chips Infected With *S. typhimurium*

For live imaging of chips, Cell Tracker green was added to chips according to manufacturer’s instructions (C2925 Thermo Fisher Scientific). Outlets and inlets were plugged with pipette tips that were cut to ¼ inch height and blocked with glue chips before imaging with a laser scanning confocal microscope (Leica SP5 X MP DMI-6000).

### Isolation of *Enterococcus* faecium From Hmb Stock

Hmb stock was plated on bile esculin agar (BEA) plates in which only Enterococcus species can hydrolize bile esculin to produce black insoluble salts. Colony PCR was performed on individual colonies. Individual colonies were inoculated into 20 ul of water and boiled at 95°C for 10 min. 16S gene was amplified using RANGER mix (Bioline Bio-25052) according to the manufacturer’s instruction and the following 16S primers 27F:  AGA GTT TGA TCM TGG CTC AG 1492R:  CGG TTA CCT TGT TAC GAC TT and PCR cycle 94°C 3 min followed by 35 cycles of: 94°C 45 s, 50°C 60 s, 72°C 90 s, ending with 72°C for 10 min. PCR product was purified using Qiaquick PCR purification kit (Qiagen 28104) and sent to Dana Farber Sequencing Core for Sanger sequencing. All sequences were identical and matched to *E. faecalis*, *E. faecium*, and *E. durans*. Growth in media containing L-Arabinose (Anaerobe Systems AS 824), Sorbitol (AS 855), or Melibiose (AS 851) revealed acid production from L-Arabinose and Melibiose indicating *E. faecium*. Growth Vancomycin Resistant Enterococcus plates (R01830 Thermo Fisher Scientific) revealed purple colonies confirming Hmb *Enterococcus* is *E. faecium*.

## Data Availability Statement

The original contributions presented in the study are publicly available. This data can be found here: BioProject, PRJNA704923.

## Ethics Statement

The animal study was reviewed and approved by Harvard Medical School Office of Institutional Animal Care and Use Committees (IACUC) Approval ID# IS00000187–3; Microbial Effect on Mammalian Host.

## Author Contributions

FGa designed and performed the experiments, analyzed data, and prepared the manuscript. DC and MW analyzed 16S data. MSP and FGr performed the experiments. AD generated *S. typhimurium mCherry*. MC and MS discussed results. DK designed experiments and discussed results. DI designed experiments, discussed results, and prepared the manuscript with FGa. All authors contributed to the article and approved the submitted version.

## Funding

This research was supported by DARPA THoR grant (W911NF-16- C-0050). MSP was financed by the Coordenação de Aperfeiçoamento de Pessoal de Nível Superior - Brasil (CAPES) - Finance Code 001.

## Conflict of Interest

DI is a founder, board member, SAB chair, and equity holder in Emulate Inc.

The remaining authors declare that the research was conducted in the absence of any commercial or financial relationships that could be construed as a potential conflict of interest.

## Supplementary Material

Supplementary Figure 1 Cells isolated from organoids derived from different regions of small intestine and colon form confluent monolayers in microfluidic Organ Chips. Organoids derived from duodenum, jejunum, ileum, and colon of C57/Bl6 mice were generated as previously described (16,17) and seeded onto microfluidic Organ Chips. Organoids from all parts of the intestine produced confluent monolayers within one week of seeding. Brightfield images of **(A)** duodenum, **(B)** jejunum, **(C)** ileum, and **(D)** colon chips 13 days after seeding.

Supplementary Figure 2 Intestinal barrier function of mouse colon chip is maintained for at least 9 days in culture. Intestinal barrier function of the mouse colon epithelium chip measured days 7–9 of chip culture by quantifying the apparent permeability (Papp) of Cascade Blue (550 Daltons).

Supplementary Figure 3 Quantification of photoconverted cells over time. Quantification of red photoconverted cells in **Figure 1D**. Red area (black line) and number of red spots (pink line) over time was quantified using FIJI2.

Supplementary Figure 4 Muc2, ChrA, and Lgr5 gene expression in sterile Colon Chips relative to expression levels in colon organoids. Sterile colon chips and organoids were maintained for 8 days before RNA extraction. *Muc2*, *ChrA*, and *Lgr5* RNA was detected in both chips and organoids, but *Muc2* and *ChrA* were expressed 98-fold and 498-fold higher, respectively in chips and *Lgr5* was expressed 10-fold higher in organoids.

Supplementary Figure 5
*S. typhimurium* visualization on-chip. Chips were infected with *S. typhimurium*-mCherry. Twenty-four hours later, *S. typhimurium* could be detected by **(A)** live microscopic imaging of *S. typhimurium* (magenta) and epithelium (green, Cell Tracker) as well as **(B)** after 4% PFA fixation (*S. typhimurium*, yellow; DAPI-stained nuclei (blue).

Supplementary Figure 6 CXCL1 and CXCL2 are released 24 h after *S. typhimurium* infection. Chips were infected with *S. typhimurium* and basal outflow was collected 1.5, 3, 6, 24 h after infection. **(A)** CXCL1 and **(B)** CXCL2 protein levels were increased 24 h after infection whereas expression levels of other cytokines **(C)** were very low.

Supplementary Figure 7 Gene expression 6 h after *S. typhimurium* infection. Mouse intestine chips were infected with *S. typhimurium* for 6 h or maintained sterile and RNAs were analyzed by microarray sequencing; differentially expressed genes with a false discovery rate of q < 0.05 are plotted.

Supplementary Figure 8 Alpha diversity of Hmb and Mmb seeding stocks and chips. Observed alpha diversity (richness) and Shannon diversity of Hmb and Mmb stocks diluted 1:10, 1:1,000, and after 40 h on chip when seeded with 1:1,000 dilution. There was a high degree of diversity within samples for microbiomes grown in the Colon Chips. When the chips were colonized with Hmb or Mmb for 16 h, and then spiked with a human *E. coli* isolate for 24 h, there was no significant impact on diversity.

Supplementary Figure 9 Isolation and identification of *E. faecium* from the Hmb stock. Hmb stock was plated on bile esculin agar (BEA) plates in which only *Enterococcus* species can hydrolyze bile esculin to produce black insoluble salts. 16S sequencing was performed on individual colonies picked from BEA plates. All sequences were identical and matched to *E. faecalis*, *E. faecium*, and *E. durans*. Growth in media containing L-Arabinose, Sorbitol, or Melibiose revealed acid production from L-Arabinose and Melibiose indicating *E. faecium*. Growth Vancomycin Resistant Enterococcus plates revealed purple colonies confirming Hmb *Enterococcus* is *E. faecium*.

Supplementary Movie S1 Colonic epithelial cells form a polarized monolayer in the mouse Colon Chip. Movie shows Z- stack images taken every 2.25 microns of sterile chip from base of membrane towards top of apical channel. Nuclei (DAPI, blue) appear at the base followed by tight junctions (ZO-1, green) and mucus (MUC2, magenta) towards the top.
