# Supplementary figures and images for: Harnessing Colon Chip Technology to Identify Commensal Bacteria That Promote Host Tolerance to Infection
Source: Front Cell Infect Microbiol. 2021 Mar 12;11:638014. doi: 10.3389/fcimb.2021.638014 (PMC7996096; doi:10.3389/fcimb.2021.638014)

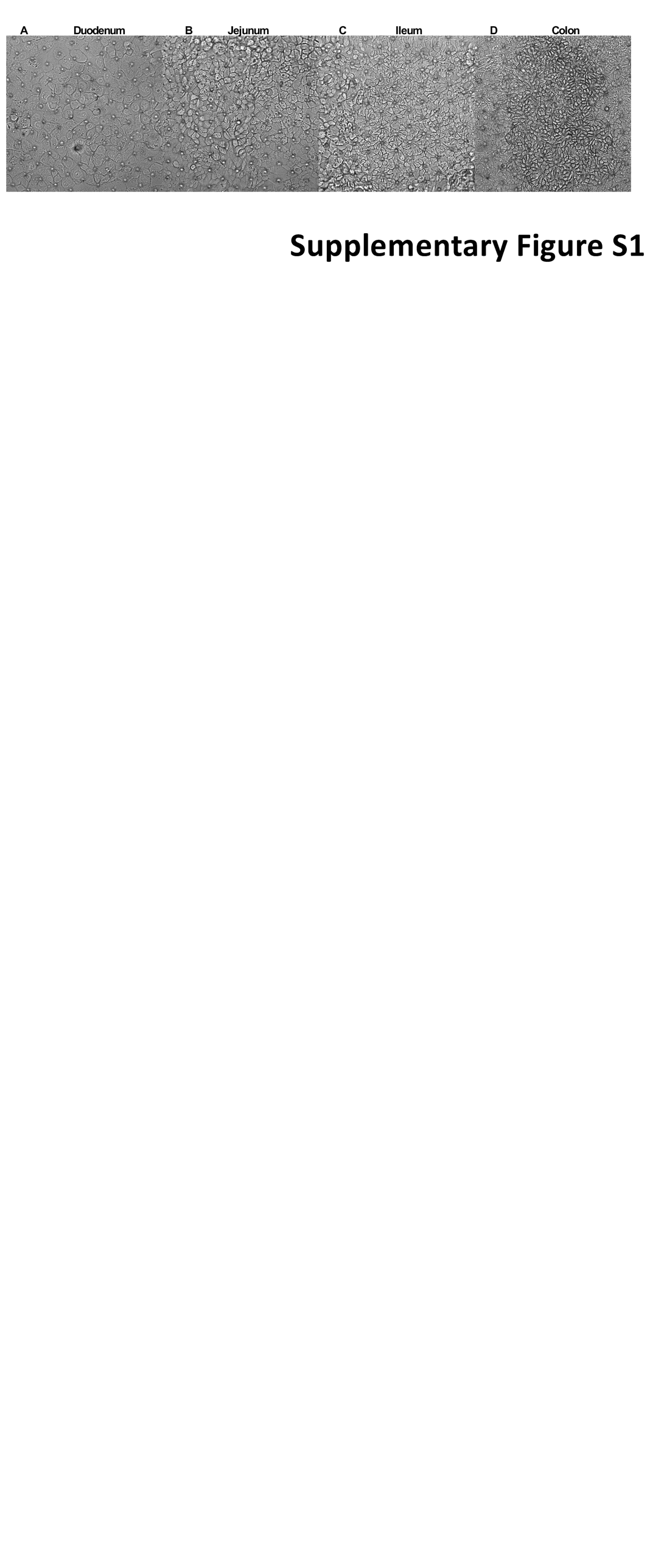

Supplement: Supplementary Figure 1 — Cells isolated from organoids derived from different regions of small intestine and colon form confluent monolayers in microfluidic Organ Chips. Organoids derived from duodenum, jejunum, ileum, and colon of C57/Bl6 mice were generated as previously described (16,17) and seeded onto microfluidic Organ Chips. Organoids from all parts of the intestine produced confluent monolayers within one week of seeding. Brightfield images of (A) duodenum, (B) jejunum, (C) ileum, and (D) colon chips 13 days after seeding. [file Image_1.jpg]

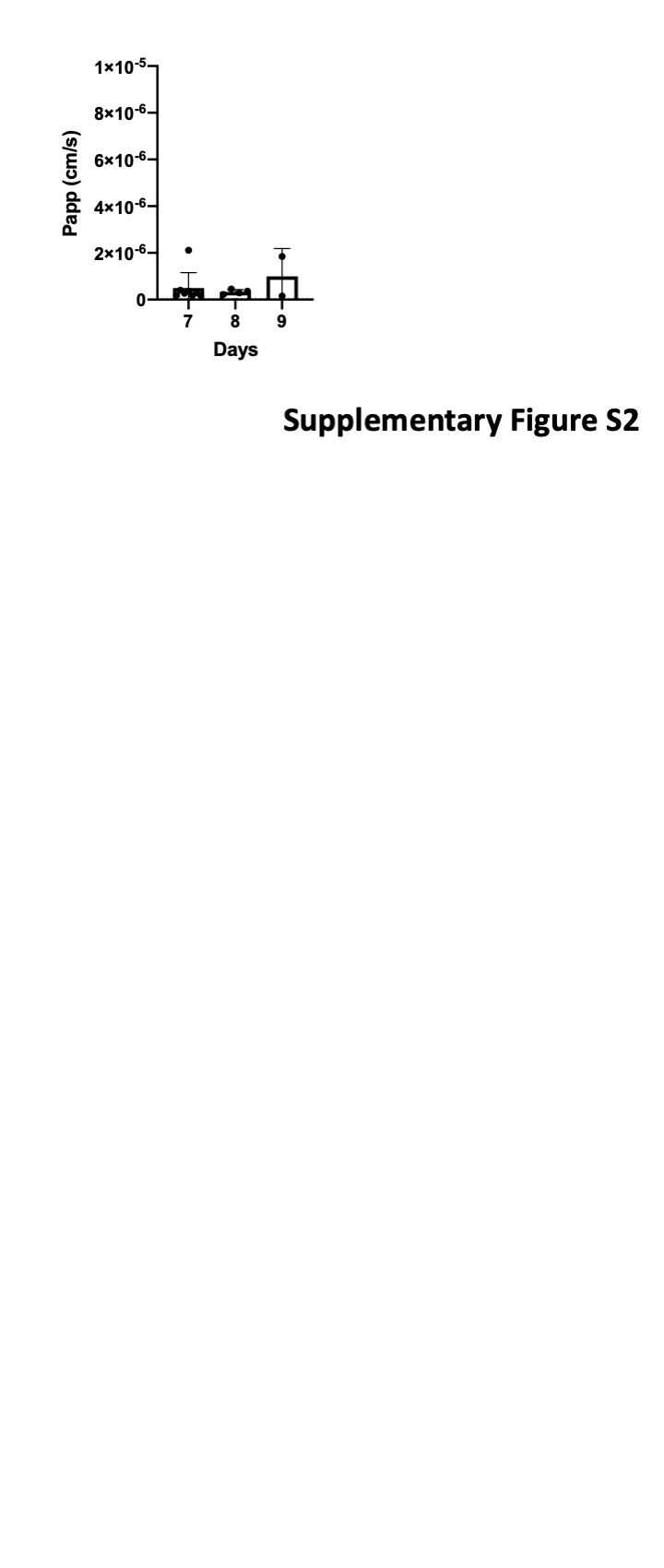

Supplement: Supplementary Figure 2 — Intestinal barrier function of mouse colon chip is maintained for at least 9 days in culture. Intestinal barrier function of the mouse colon epithelium chip measured days 7–9 of chip culture by quantifying the apparent permeability (Papp) of Cascade Blue (550 Daltons). [file Image_2.jpeg]

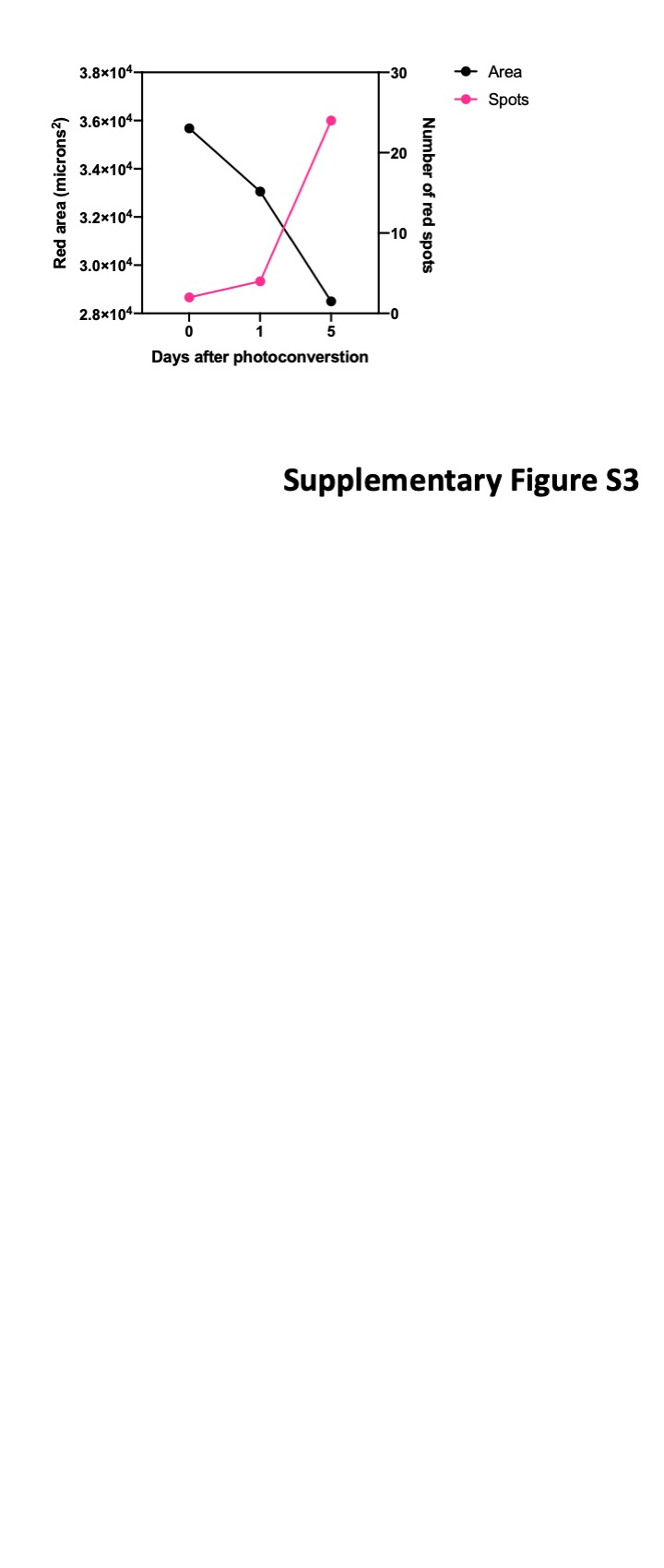

Supplement: Supplementary Figure 3 — Quantification of photoconverted cells over time. Quantification of red photoconverted cells in Figure 1D . Red area (black line) and number of red spots (pink line) over time was quantified using FIJI2. [file Image_3.jpeg]

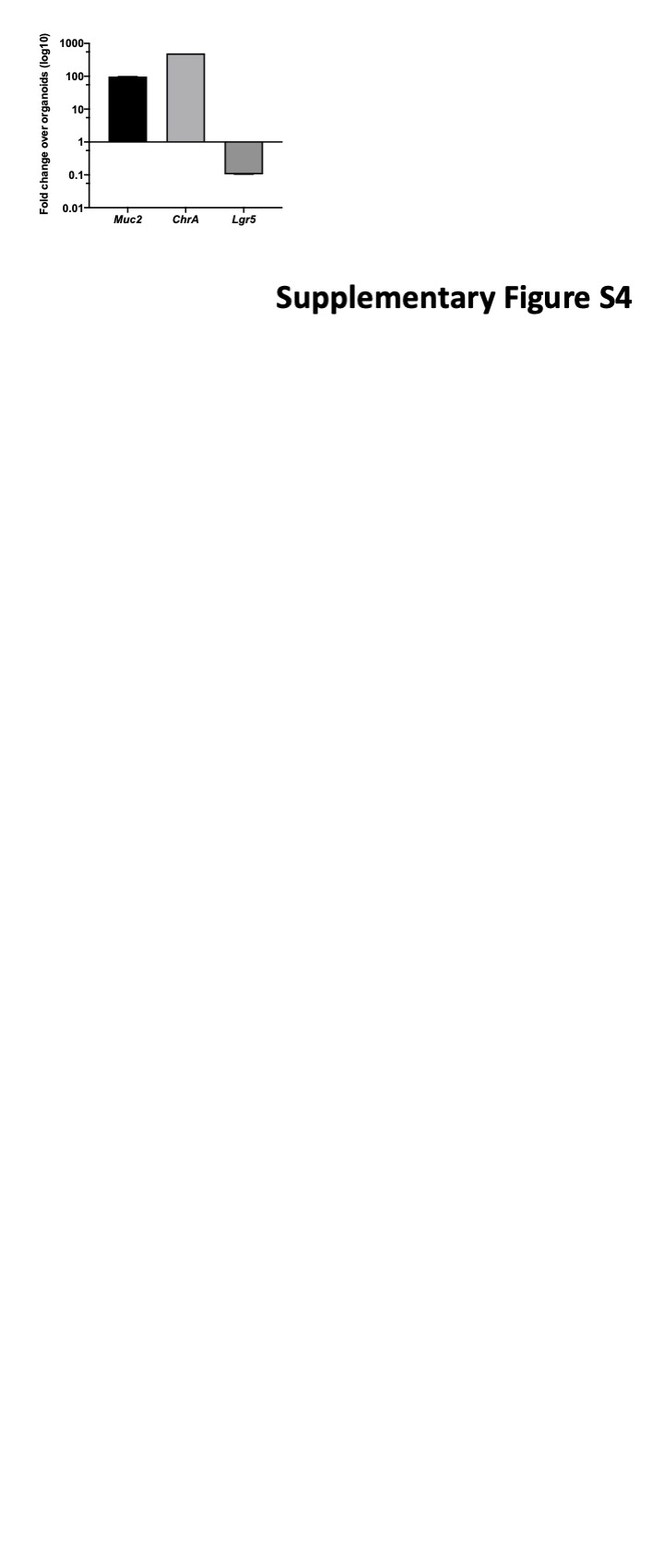

Supplement: Supplementary Figure 4 — Muc2, ChrA, and Lgr5 gene expression in sterile Colon Chips relative to expression levels in colon organoids. Sterile colon chips and organoids were maintained for 8 days before RNA extraction. Muc2, ChrA, and Lgr5 RNA was detected in both chips and organoids, but Muc2 and ChrA were expressed 98-fold and 498-fold higher, respectively in chips and Lgr5 was expressed 10-fold higher in organoids. [file Image_4.jpeg]

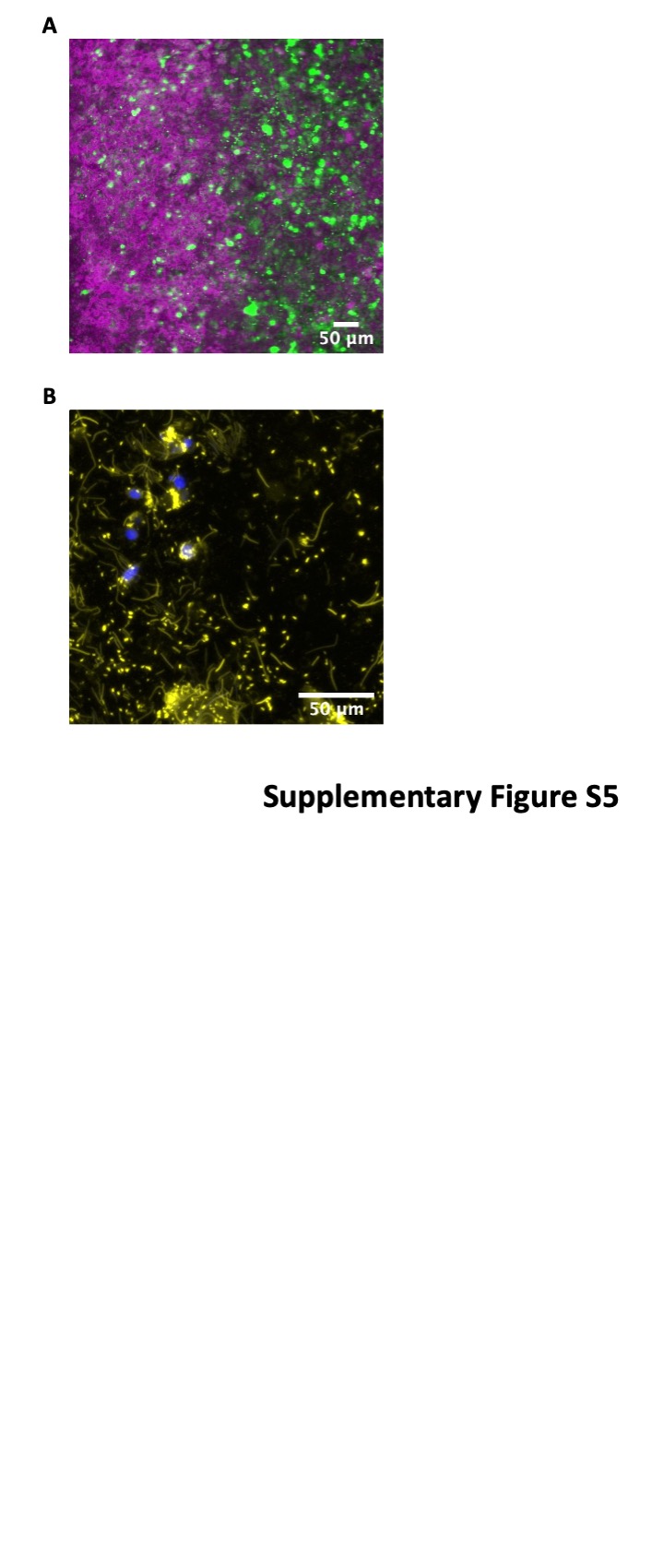

Supplement: Supplementary Figure 5 — S. typhimurium visualization on-chip. Chips were infected with S. typhimurium-mCherry. Twenty-four hours later, S. typhimurium could be detected by (A) live microscopic imaging of S. typhimurium (magenta) and epithelium (green, Cell Tracker) as well as (B) after 4% PFA fixation (S. typhimurium, yellow; DAPI-stained nuclei (blue). [file Image_5.jpeg]

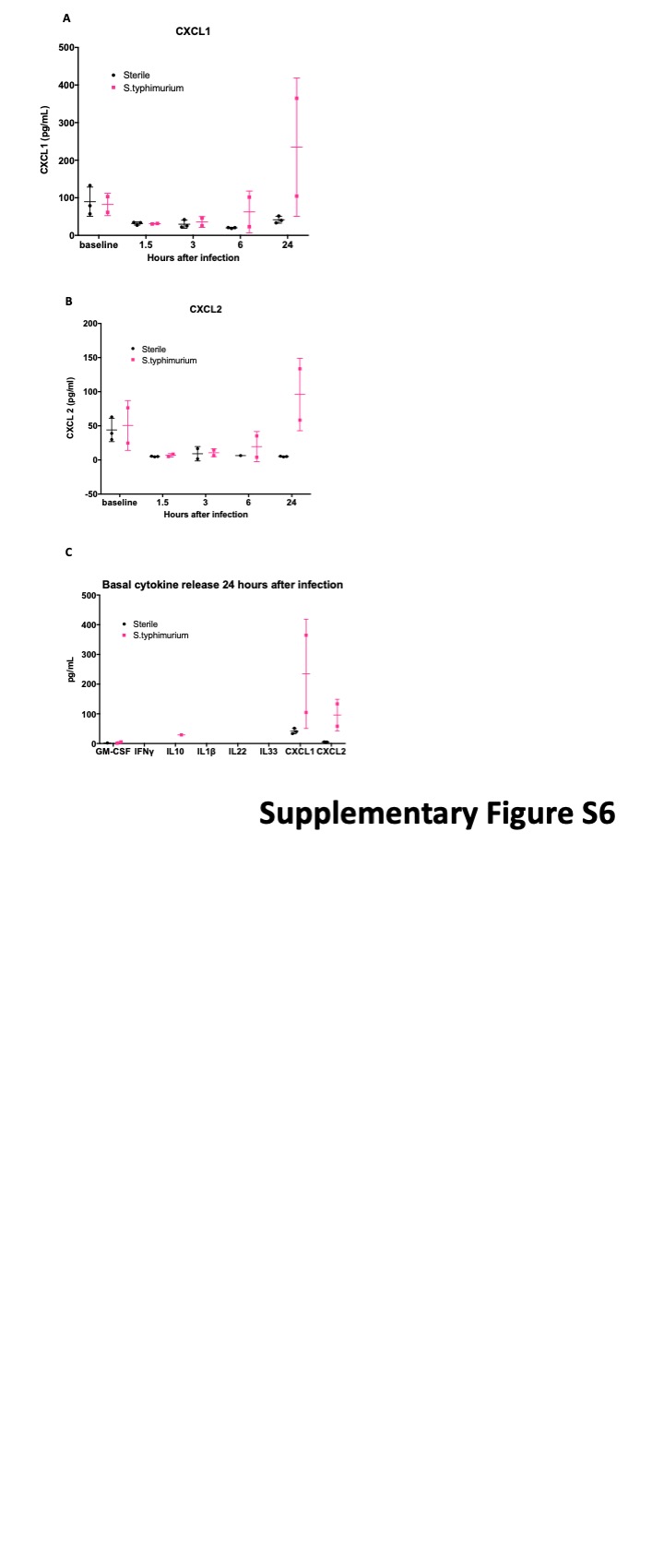

Supplement: Supplementary Figure 6 — CXCL1 and CXCL2 are released 24 h after S. typhimurium infection. Chips were infected with S. typhimurium and basal outflow was collected 1.5, 3, 6, 24 h after infection. (A) CXCL1 and (B) CXCL2 protein levels were increased 24 h after infection whereas expression levels of other cytokines (C) were very low. [file Image_6.jpeg]

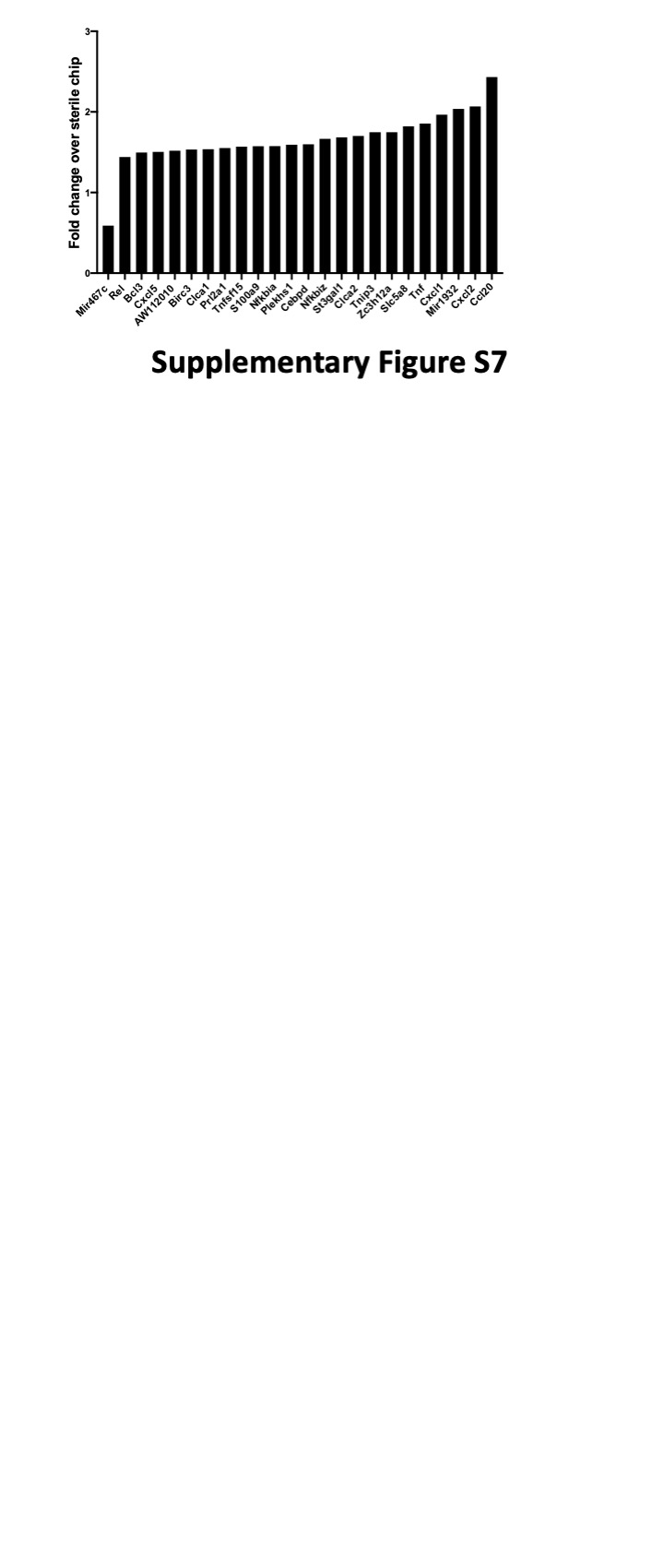

Supplement: Supplementary Figure 7 — Gene expression 6 h after S. typhimurium infection. Mouse intestine chips were infected with S. typhimurium for 6 h or maintained sterile and RNAs were analyzed by microarray sequencing; differentially expressed genes with a false discovery rate of q < 0.05 are plotted. [file Image_7.jpeg]

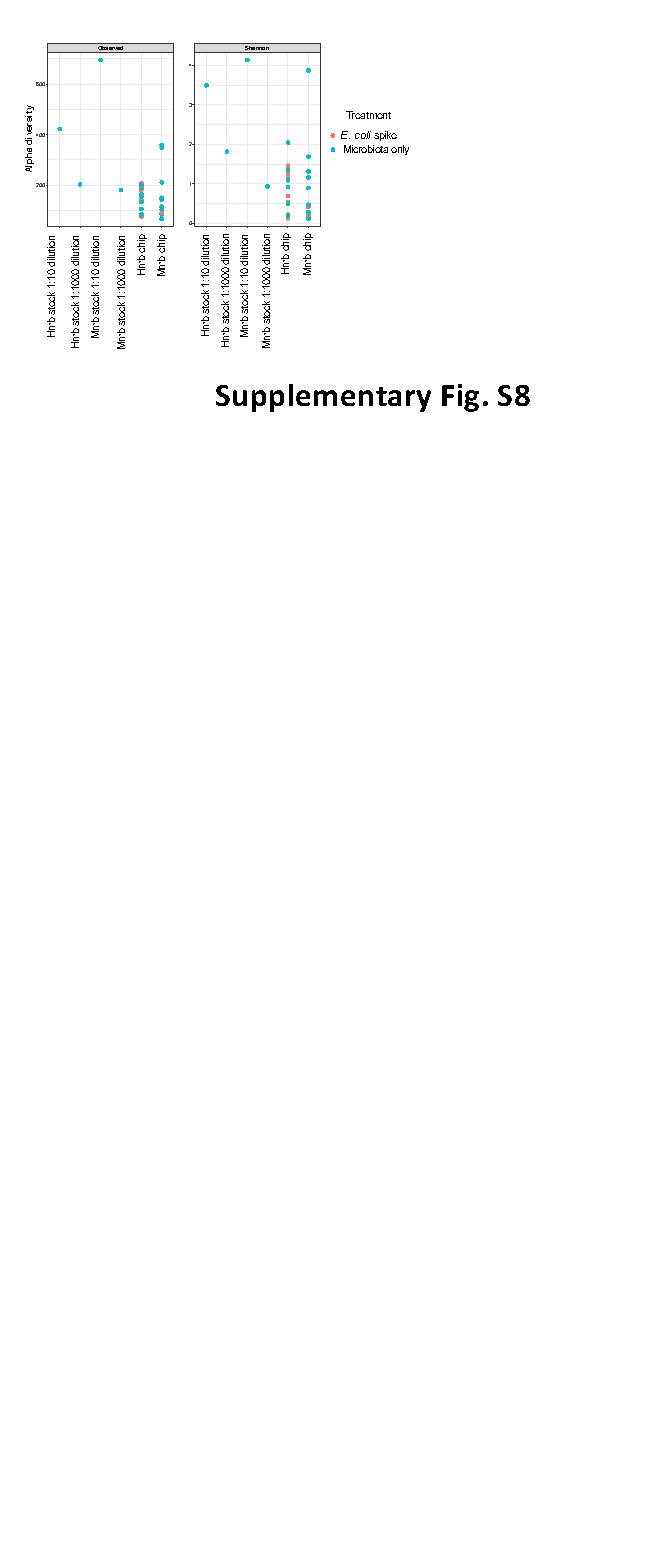

Supplement: Supplementary Figure 8 — Alpha diversity of Hmb and Mmb seeding stocks and chips. Observed alpha diversity (richness) and Shannon diversity of Hmb and Mmb stocks diluted 1:10, 1:1,000, and after 40 h on chip when seeded with 1:1,000 dilution. There was a high degree of diversity within samples for microbiomes grown in the Colon Chips. When the chips were colonized with Hmb or Mmb for 16 h, and then spiked with a human E. coli isolate for 24 h, there was no significant impact on diversity. [file Image_8.jpg]

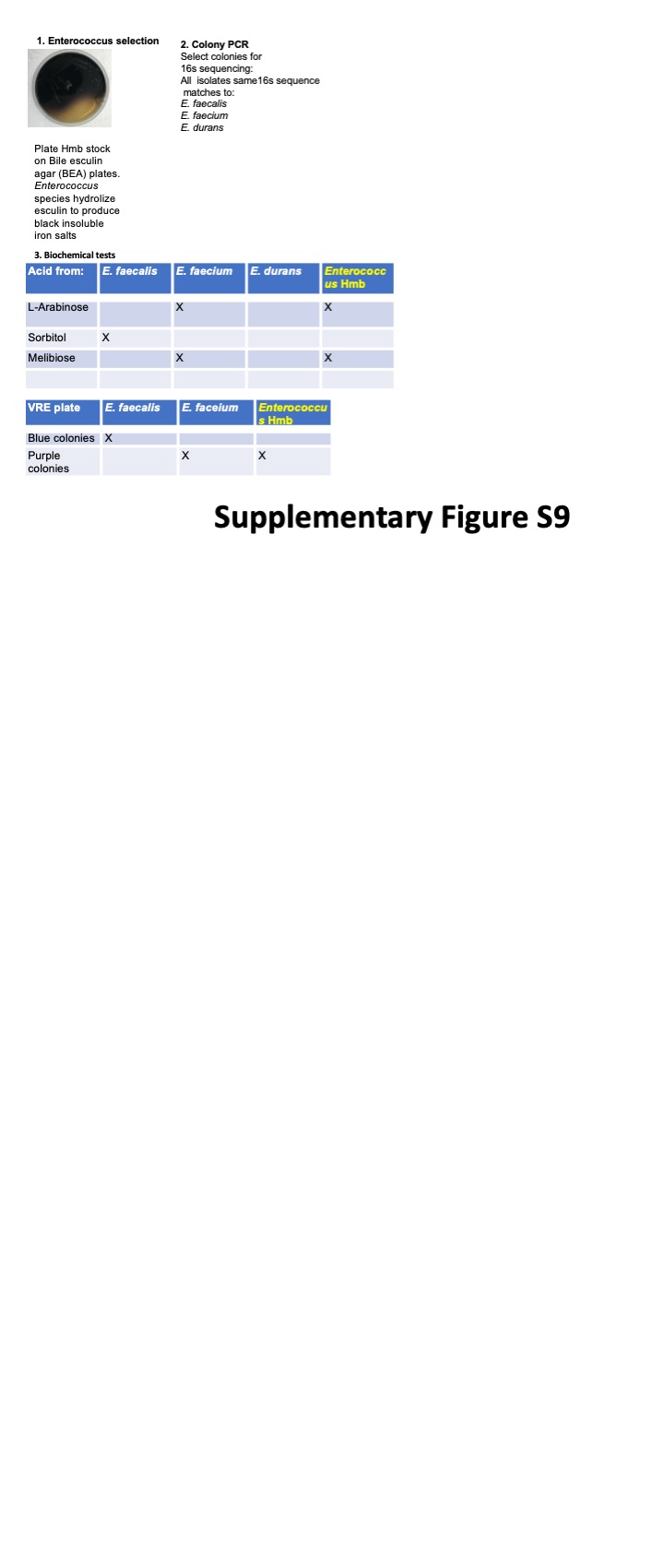

Supplement: Supplementary Figure 9 — Isolation and identification of E. faecium from the Hmb stock. Hmb stock was plated on bile esculin agar (BEA) plates in which only Enterococcus species can hydrolyze bile esculin to produce black insoluble salts. 16S sequencing was performed on individual colonies picked from BEA plates. All sequences were identical and matched to E. faecalis, E. faecium, and E. durans. Growth in media containing L-Arabinose, Sorbitol, or Melibiose revealed acid production from L-Arabinose and Melibiose indicating E. faecium. Growth Vancomycin Resistant Enterococcus plates revealed purple colonies confirming Hmb Enterococcus is E. faecium. [file Image_9.jpeg]
